# Between Screens and Self-Perception: The Role of Gender and Digital Media Use in Shaping Body Esteem and Self-Esteem Among Adolescents

**DOI:** 10.3390/children12091143

**Published:** 2025-08-28

**Authors:** Mateusz Grajek, Tomasz Jurys, Mateusz Rozmiarek

**Affiliations:** 1 Department of Public Health, Faculty of Public Health in Bytom, Medical University of Silesia in Katowice, 41-902 Bytom, Poland; 2 Department of Rehabilitation, Faculty of Health Sciences in Katowice, Medical University of Silesia in Katowice, 40-751 Katowice, Poland; tomasz.jurys@sum.edu.pl; 3 Department of Sports Tourism, Faculty of Physical Culture Sciences, Poznan University of Physical Education, 61-871 Poznan, Poland; rozmiarek@awf.poznan.pl

**Keywords:** adolescents, body esteem, self-esteem, internet use, gender differences

## Abstract

**Highlights:**

**What are the main findings?**

- Girls reported significantly lower body esteem and self-esteem than boys, despite similar BMI levels.
- Prolonged Internet use (especially over 4 h daily) was negatively associated with body and global self-esteem, particularly among girls.

**What is the implication of the main finding?**

- Digital media exposure is a stronger predictor of adolescent self-perception than objective body measures like BMI.
- Targeted prevention strategies should address gender-specific vulnerabilities and promote healthy digital habits in adolescents.

**Abstract:**

Background/Objectives: Adolescence is a critical period for identity development and self-perception, increasingly shaped by digital media. This study aimed to examine how gender, body mass index (BMI), and Internet use influence body esteem and global self-esteem among adolescents aged 15–18 years old, with particular focus on the psychological impact of digital exposure. Methods: A three-wave online study was conducted using the Computer-Assisted Web Interview (CAWI) technique. The final sample consisted of 500 Polish adolescents (251 girls, 249 boys). Participants completed the Body Esteem Scale (BES) and Rosenberg Self-Esteem Scale (SES) at three time points. The study explored gender differences, the role of BMI, and the impact of time spent online. Results: Girls reported significantly lower BES and SES scores than boys (*p* < 0.001), despite no significant gender differences in BMI. Time spent online was negatively associated with both body esteem and self-esteem (*p* < 0.01), with the most pronounced effects among girls using the Internet for more than 4 h daily. Regression analyses identified gender and Internet use as significant predictors of self-perception, while BMI did not. Conclusions: Digital media use, particularly prolonged exposure, appears to be a stronger predictor of adolescent self-perception than objective body measures such as BMI. Girls are especially vulnerable to its adverse effects on both body esteem and global self-esteem. These findings underscore the need for gender-sensitive interventions focused on media literacy, emotional resilience, and healthy digital habits among adolescents.

## 1. Introduction

In the digital age, adolescence unfolds in an environment increasingly shaped by ubiquitous access to the Internet and social media platforms [1]. Unlike previous generations, today’s youth construct their identities, navigate peer relationships, and engage in self-presentation not only in physical spaces but also across complex digital landscapes [2]. Social media have evolved beyond tools of communication to become mirrors of cultural norms, arenas of comparison, and stages for performing idealized versions of the self [3,4]. These platforms offer opportunities for creativity and connection, yet simultaneously expose adolescents to curated imagery, algorithmic feedback, and constant visibility—factors that may profoundly influence their sense of self [5]. As such, digital environments have become central to the construction and experience of self-perception among adolescents.

Adolescence is a developmental stage marked by heightened sensitivity to self-image, social approval, and belonging [6]. Two constructs central to this period are body esteem—the evaluative perception of one’s body and physical appearance—and global self-esteem, or the broader sense of self-worth [7,8]. These components of self-perception play a pivotal role in adolescent well-being, influencing emotional resilience, interpersonal functioning, and susceptibility to disorders such as depression, anxiety, and body dysmorphia [9]. A positive self-image during this stage can serve as a protective buffer, whereas negative evaluations may lead to enduring psychological distress. Given the developmental importance of these constructs, investigating how contemporary media environments intersect with individual differences to shape them is of increasing relevance.

A growing body of empirical research highlights the significant role of digital media in shaping adolescents’ self-perception, particularly through gendered mechanisms of appearance-related comparison and self-presentation [10,11,12]. Social media platforms are saturated with idealized imagery that promotes narrow beauty standards—slimness, muscularity, flawless skin—which are often enhanced through filters, editing tools, and algorithmic curation [13]. Adolescents exposed to this content may engage in upward social comparisons, internalizing unrealistic ideals that contribute to body dissatisfaction, lower body esteem, and diminished global self-worth. While girls are more frequently targeted by and engaged with beauty-centric content—making them especially vulnerable to appearance-related pressures [14]—boys are not immune. Increasingly, adolescent boys report pressures associated with muscularity, athletic appearance, and strength, perpetuated by fitness influencers, avatars in gaming culture, and sports media [15]. Moreover, these media experiences intersect with individual differences such as body mass index (BMI), where adolescents with higher BMI may be at greater risk of negative self-evaluation [16]. Gender and BMI thus function as key moderators in how adolescents consume, interpret, and are affected by digital media, influencing not only the types of content they engage with, but also the psychological impact of that engagement.

While much of the literature on adolescent self-perception and media influence has been conducted in Western contexts, emerging studies from Asia and Latin America confirm the global relevance of these dynamics as well. For instance, research from South Korea and China has documented strong associations between social media exposure and appearance-related pressures among adolescents, highlighting cultural similarities in vulnerability despite differing sociocultural ideals. Incorporating such perspectives broadens the scope of the present study and underscores its contribution to understanding adolescent well-being in a digitally interconnected world [17,18].

Given these complexities, this study aims to investigate how gender, BMI, and patterns of digital media use collectively influence body esteem and global self-esteem among adolescents aged 15–18 years old. Despite growing attention to these issues, few studies have integrated these variables within a single analytical framework or focused specifically on older adolescents—a group at a pivotal juncture in identity development and media engagement. This research seeks to address that gap by examining how sociocultural, behavioral, and psychological dimensions interact in shaping adolescent self-perception. Through this lens, we aim to contribute to a more nuanced understanding of adolescent well-being in the digital age and offer evidence to inform educational, clinical, and policy interventions.

## 2. Objective

The primary aim of this study was to examine gender differences in body esteem and global self-esteem among adolescents aged 15–18 years old, while identifying the predictive role of daily Internet use and body mass index (BMI). Particular attention was given to patterns of digital media consumption and their associations with psychological well-being, with the goal of exploring how environmental and perceptual factors interact in shaping adolescent self-perception.

We formulated the following hypotheses: (1) adolescent girls will report significantly lower body esteem and global self-esteem than boys, despite similar BMI levels; (2) prolonged daily Internet use will be negatively associated with body esteem and self-esteem, with stronger effects among girls; and (3) BMI will show weaker associations with self-perception compared to digital media use.

## 3. Materials and Methods

### 3.1. Research Procedure

The study was conducted using a three-wave measurement design, employing the CAWI (Computer-Assisted Web Interviewing) technique for online data collection. The data were gathered between February and May 2025 via a secure, dedicated research platform that complied with the technical requirements and the General Data Protection Regulation (GDPR; EU Regulation 2016/679).

Each participant was assigned a randomly generated, individual identification code (an 8-character alphanumeric string), which was required to be re-entered at each wave (T1, T2, T3) to ensure data consistency. This code did not permit personal identification but served solely to link responses across time points. Measurements were conducted at two-week intervals. As a result, out of the initial pool of 528 individuals who completed at least one questionnaire, a final sample of 500 participants was retained for analysis. This group provided three complete and internally consistent responses, all linked to the same identifier. The remaining 28 cases were excluded due to incomplete data or inconsistencies in the identifier (e.g., mismatched codes across waves).

Post-hoc power analyses indicated that with a sample size of 500, the study had over 95% power to detect medium-sized effects (Cohen’s d = 0.5; η^2^ = 0.06) at α = 0.05, confirming the adequacy of the sample for the reported analyses.

### 3.2. Research Participants

Inclusion criteria were as follows: age between 15 and 18 years old at the time of the first measurement, informed consent to participate (with additional parental or legal guardian consent for minors), fluency in Polish, and regular access to the Internet and a device capable of independently completing an online survey (e.g., computer, tablet, or smartphone). Exclusion criteria included: age outside the designated range, inability to participate in all three waves, incomplete data, unreliable responses (e.g., extreme intraindividual inconsistencies), and lack of parental consent in the case of underage participants.

The final sample consisted of 500 adolescents (251 girls and 249 boys) whose data were complete, logically coherent, and consistently linked by the same identification code across all measurement points. Characteristics of the participants are presented in Table 1.

### 3.3. Research Tools

Body Esteem Scale (BES)—The version adapted for adolescent populations was used. The scale consists of 20 items rated on a five-point Likert scale (1–5), with higher scores indicating greater body acceptance. The BES demonstrated high internal consistency in the current study (Cronbach’s α = 0.91), good temporal stability (test-retest r = 0.83), and strong convergent validity with measures of self-esteem and social anxiety.

Rosenberg Self-Esteem Scale (SES)—The widely used 10-item self-report scale was employed to assess global self-esteem. Responses were recorded on a four-point Likert scale (ranging from “strongly disagree” to “strongly agree”). In this study, the SES exhibited good internal consistency (Cronbach’s α = 0.87) and satisfactory test-retest reliability (r = 0.79).

For each participant, final scores were computed as the arithmetic mean across the three time points (T1, T2, T3), allowing for the reduction of transient mood effects and improving the validity of the assessment of stable cognitive-affective constructs.

### 3.4. Research Ethics

The study adhered to the principles outlined in the Declaration of Helsinki and the guidelines of the European Federation of Psychologists’ Associations (EFPA). The research protocol was approved by the Bioethics Committee (BNW/NWN/0052/KB/171/24 19.11.2024). Participants were assured full anonymity and informed of their right to withdraw at any stage. All respondents (or their legal guardians, where applicable) provided informed consent after being presented with the study’s objectives and data processing procedures.

### 3.5. Statistical Analysis

Statistical analyses were performed using Statsoft Statistica 13.3. Independent samples *t*-tests, one-way analysis of variance (ANOVA), Tukey’s HSD post hoc tests, and ordinary least squares (OLS) linear regression were applied. A significance level of *p* < 0.05 was adopted, with Bonferroni correction used where appropriate to control for multiple comparisons.

## 4. Results

A total of 500 adolescents aged 15 to 18 years old participated in the study (M = 16.4, SD = 1.1), comprising 251 girls (50.2%) and 249 boys (49.8%). The mean BMI for the total sample was 22.1 (SD = 3.0). No significant gender differences in BMI were observed, t(498) = 0.79, *p* = 0.43, suggesting that gender-related differences in body image cannot be attributed to disparities in body proportions across groups. Regarding Internet use, a significantly higher proportion of girls than boys reported spending more than 4 h online daily (53.7% vs. 38.6%), confirmed by a chi-square test, χ^2^(1) = 10.72, *p* = 0.001.

### 4.1. Internet Activity of Participants

Although this study revealed gendered differences in online activity patterns, it did not distinguish specific content categories (e.g., beauty influencers, fitness tutorials, educational channels). Future research should incorporate more granular distinctions, as different types of media may exert unique psychological effects. Based on data collected through a custom-designed questionnaire on Internet activity, participants were asked to indicate the types of content or platforms they most frequently engage with online (multiple-choice question). Respondents could select more than one option, with the question referring specifically to daily habits rather than occasional usage. The analysis revealed clear patterns of preference among adolescents, with social media emerging as the dominant category. The most frequently reported form of online activity was social media use—including platforms such as TikTok, Instagram, Snapchat, and Facebook—endorsed by 86.4% of all participants. A gender-stratified analysis showed that 92.0% of girls and 80.7% of boys selected this category, representing a statistically significant difference (χ^2^(1) = 14.21, *p* < 0.001). Girls were also more active on visually-oriented and appearance-focused platforms (e.g., Instagram, TikTok), whereas boys more often reported using information- and communication-oriented applications such as Discord and Reddit. The second most popular category was video streaming services (e.g., YouTube, Netflix), used daily by 68.2% of participants, with no significant gender differences (girls: 66.9%, boys: 69.5%). Online gaming and gaming-related platforms (e.g., Twitch, Steam, Discord) ranked third, selected by 53.8% of the overall sample. However, a notable gender disparity emerged: 70.3% of boys versus only 37.1% of girls reported regular gaming activity (χ^2^(1) = 61.74, *p* < 0.001). News and information websites were accessed by 28.4% of respondents, with slightly higher rates among boys (31.7%) than girls (25.1%), though this difference did not reach statistical significance. A smaller subgroup (17.6%) reported daily use of educational or academic websites, with a slight female predominance (girls: 20.3%, boys: 14.8%). Importantly, nearly all participants (98.4%) reported daily Internet use via mobile devices, which correlates with the rising consumption of fast, image-based, and short-form content—characteristic of social media platforms. Taken together, these findings clearly indicate that social media constitutes the central domain of adolescents’ digital activity, particularly among girls. In contrast, boys’ online behavior appears more diversified, encompassing not only social networking but also gaming and informational content. These gender-specific media consumption patterns should be carefully considered in future analyses examining the impact of digital environments on self-esteem, body image, and psychological well-being in adolescence.

### 4.2. BES and SES Evaluation

Analysis of the BES scores revealed significant gender differences. Girls scored lower on average (M = 42.06, SD = 9.81) than boys (M = 46.84, SD = 9.81), with the difference reaching statistical significance, t(498) = 5.13, *p* < 0.001. The effect size (Cohen’s d = 0.49) indicates a medium magnitude, aligning with existing literature that adolescent females tend to report lower body acceptance than their male peers. To examine the effect of BMI on BES scores, a one-way ANOVA was conducted across four BMI categories (underweight, normal weight, overweight, obesity). The results were not statistically significant, F(3, 496) = 1.87, *p* = 0.133, with a small effect size (η^2^ = 0.011), indicating that objective body mass had minimal influence on body image evaluation. This supports the notion that adolescents’ perceptions of their appearance are relatively independent of somatic indicators. In contrast, time spent online significantly affected BES scores, F(3, 496) = 9.45, *p* < 0.001, η^2^ = 0.054. Participants who spent less than 2 h online daily reported the highest BES scores (M = 46.90), while those in the 4–6 h range had the lowest (M = 43.01). Post-hoc Tukey HSD tests confirmed significant differences, particularly between the <2 h and 4–6 h groups (*p* < 0.001) and between <2 h and >6 h (*p* = 0.01). Further interaction analysis revealed that the negative impact of prolonged Internet use on body esteem was more pronounced among girls: in the 4–6 h group, their mean BES score was 40.5 (SD = 8.9), compared to 47.6 (SD = 8.2) for boys in the same category. As for global self-esteem, measured via the SES, a significant gender difference was also observed. Girls scored lower (M = 21.51, SD = 4.0) than boys (M = 23.76, SD = 3.9), t(498) = 6.02, *p* < 0.001, with an effect size of d = 0.58, again indicating a medium effect of gender on self-esteem. No significant relationship between BMI and SES was found, F(3, 496) = 1.44, *p* = 0.231. However, time spent online significantly predicted SES scores, F(3, 496) = 7.02, *p* < 0.001, η^2^ = 0.041. A clear downward trend in SES scores was observed with increasing online time; participants in the >6 h group averaged 21.9 points, with the decline most pronounced among girls (M = 20.35, SD = 3.8).

Interestingly, among girls, the gap in low self-esteem and body esteem scores between the 4–6 h and >6 h groups was marginal. This may indicate a plateau effect, whereby the psychological burden of digital exposure stabilizes after a certain threshold. Alternatively, adolescents may engage in compensatory coping strategies or become desensitized after prolonged exposure. Further analysis indicated that among girls spending 4–6 h online daily, 41.8% scored below 40 on the BES—indicating low body esteem—and 34.1% scored below 20 on the SES, qualifying as low self-esteem. In contrast, among boys in the same time-use category, these proportions were notably lower: 16.2% and 13.7%, respectively. In the group spending less than 2 h online per day, only 18% of girls had low BES scores, and just 11% had low self-esteem. These findings highlight a substantial digital exposure effect, particularly detrimental to young females (Table 2).

The observed effect sizes correspond to differences of approximately half a standard deviation between girls and boys in both body esteem and self-esteem. In practical terms, this means that a substantial proportion of adolescent girls report levels of self-perception that would be considered clinically low compared to their male peers, underscoring the real-world significance of these findings.

**Figure 1:**
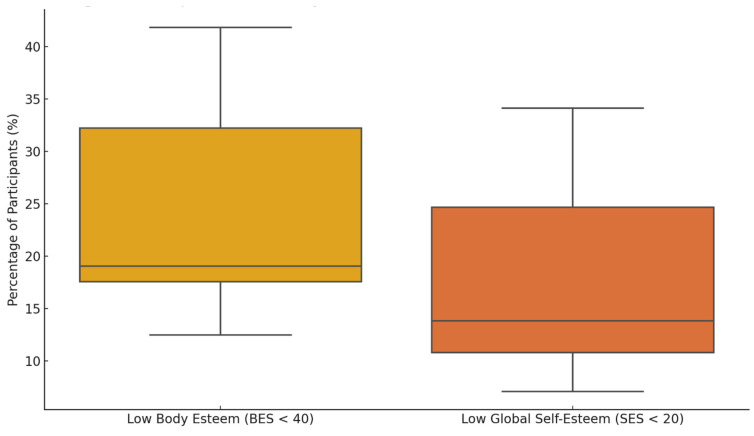
Boxplot of low body esteem and global self-esteem in adolescents.

Figure 1 presents the spread of participants reporting low body esteem and self-esteem, aggregated across gender and time-spent-online groups. The mean percentage of adolescents with low BES was 24.5% (SD = 10.5), while for low SES it was 17.9% (SD = 9.2). Female participants exhibited both a higher median and greater variability in both indicators compared to males, particularly for body esteem. The 4–6 h daily Internet use group showed the most pronounced effect, where 41.8% of girls and 16.2% of boys scored below the BES threshold, and 34.1% of girls versus 13.7% of boys scored below the SES threshold. These findings underscore a heightened psychological vulnerability among adolescent girls, especially in contexts of prolonged digital exposure.

To identify predictors of BES scores, a multiple regression analysis was conducted with gender, BMI, and daily Internet use (in hours) as independent variables. The model was statistically significant, R^2^ = 0.15; F(3, 496) = 29.41, *p* < 0.001. Gender emerged as the strongest predictor: being female was associated with a decrease of 4.57 points in BES (B = −4.57, SE = 0.90, t = −5.10, *p* < 0.001). Daily Internet use also had a significant negative effect (B = −0.56, SE = 0.22, t = −2.52, *p* = 0.012). BMI did not significantly predict BES (B = −0.11, SE = 0.15, *p* = 0.472). These results indicate that adolescent body image is more strongly influenced by psychosocial variables, particularly gender and digital exposure, than by objective body size. The same was done in the cases of SES. The model was statistically significant (R^2^ = 0.11, F(3, 496) = 21.32, *p* < 0.001). Gender emerged as the strongest predictor: being female lowered SES scores by an average of 2.22 points (B = −2.22, SE = 0.35, t = −6.31, *p* < 0.001). Daily hours online also exerted a significant negative effect (B = −0.40, SE = 0.09, t = −4.58, *p* < 0.001). BMI was not a significant predictor (*p* = 0.69) (Table 3).

**Figure 2:**
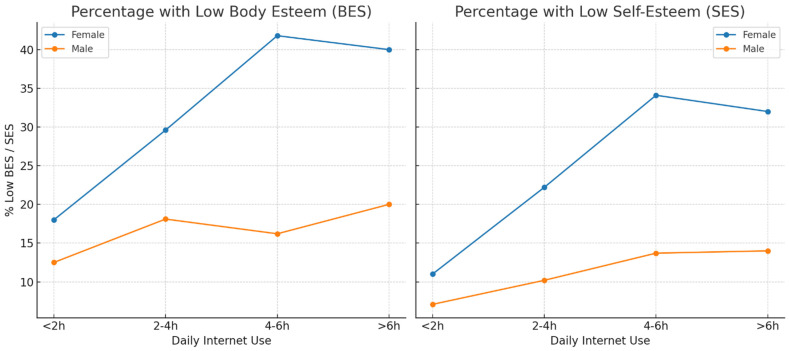
Line plots of mean BES and SES scores across daily Internet use categories, stratified by gender.

Additionally, the regression model including the interaction term Gender × Daily Internet Use revealed a significant interaction effect for both body esteem (B = −0.32, SE = 0.11, *p* = 0.004) and self-esteem (B = −0.21, SE = 0.08, *p* = 0.012). These results indicate that the negative association between prolonged Internet use and self-perception was significantly stronger among girls than among boys. Figure 2 illustrates the downward trend in BES and SES scores with increasing daily Internet use, with steeper declines observed among girls than boys, further visualizing the interaction effects.

## 5. Discussion

The results of the study indicate clear gender differences in global self-esteem and body acceptance among adolescents aged 15–18 years old. Girls scored significantly lower on both the Rosenberg Scale (SES) and the BES scale, which is consistent with previous findings in the literature, according to which adolescent girls are more susceptible to negative perceptions of their own bodies than their male peers [19,20,21]. In the study by Gomez-Baya et al., results shows that girls present lower self-esteem (U = 323,222.50, Z = −7.10, *p* < 0.001, MD = 1.64), also lower perceived emotional clarity (U = 376,161.50, Z = −2.26, *p* = 0.024, MD = 0.35), as well as lower perceived emotional repair (U = 329,596.50, Z = −6.53, *p* < 0.001, MD = 1.28) [20]. Also, girls reported low self-esteem more often in the study by Carlén et al., which shows a difference −2.168 (*p* < 0.001) at ages 12–13 years old and—2.712 (*p* < 0.001) at the age of 17 years old [21]. Both studies also indicate that lower self-esteem could be a strong predictor of a decrease in mental well-being [16,17,18].

Another important finding was that these differences were not related to objective indicators of body composition—BMI did not differ significantly between groups and was not a predictor of either BES or SES. Different conclusions were presented in a study by Rentz-Fernandes et al., involving 418 young people aged 14–18 years old [22]. The mentioned study shows that boys exhibited a higher prevalence of overweight or obesity, but reported lower levels of depression and body dissatisfaction, along with higher self-esteem, compared to girls (*p* < 0.001). Depression was negatively associated with self-esteem (*p* < 0.01), and self-esteem was inversely related to body dissatisfaction (*p* < 0.01). Interestingly, adolescents with better nutritional status were more likely to experience body dissatisfaction (*p* < 0.001). However, BMI was associated with body dissatisfaction only among girls (*p* < 0.01). The relationship between depression and body dissatisfaction differed by gender: it was negative for boys (*p* < 0.01) and positive for girls (*p* < 0.01) [22]. In turn, research conducted by Petrovics et al. on a group of nearly 400 students indicates no significant correlation, as the BMI variable yielded a β coefficient of 0.07, which indicates a positive but statistically insignificant relationship between BMI and self-esteem [23]. This means that as BMI increases for a given age, self-esteem may increase slightly, but this effect does not reach statistical significance (*p* > 0.05) [23].

One of the most important findings of the study is the confirmation of the negative impact of time spent online on self-esteem and body acceptance, especially among girls. The more hours per day adolescent girls spent on the Internet (especially in the 4–6 h range), the more often they reported low self-esteem and dissatisfaction with their appearance. In line with findings shown by Yang et al., on social media use, we observed a significant indirect effect of overall smartphone screen time on body esteem only among participants who reported using their smartphones for more than 4 h per day. Specifically, excessive smartphone use significantly increased the cognitive internalization of an ideal body image (B = 0.455, SE = 0.232, *p* = 0.05), which in turn heightened engagement in appearance comparisons (B = 1.422, SE = 0.151, *p* < 0.001). That led to increased social appearance anxiety (B = 0.142, SE = 0.049, *p* = 0.005), ultimately resulting in lower body esteem (B = −0.307, SE = 0.056, *p* < 0.001) [24]. This effect was not observed to the same intensity in boys, suggesting that girls are more sensitive to visual and social triggers present in the digital environment, including beauty standards promoted on social media. These results are partially confirmed by findings from the study by Twenge and Farley, where among adolescents using social media for more than 5 h per day (compared to less than 1 h), girls were 88% more likely to report low self-esteem (aRRR = 1.88), whereas the increase for boys was 35% (aRRR = 1.35). This gender difference was statistically significant (Z = 2.21, *p* < 0.05) [25]. It can be explained by girls using apps that focus on visual presentation (like Instagram and TikTok) more often, which can lead to social comparisons and internalizing unrealistic beauty standards.

Gender and time spent online had a significant and negative impact on both psychological indicators. These results suggest that the mental health of young people today is shaped more by the digital environment and its nature than by objective biological factors such as body weight. The present finding is consistent with previous research indicating that subjective perceptions of one’s own body may be more dependent on social comparisons and cultural pressures than on actual somatic characteristics. In the study by Vuong et al., it was found that social media use, thin-ideal internalization, and muscular-ideal internalization were all positively and significantly associated with body dissatisfaction (rs = 0.10, *p* < 0.05; rs = 0.24, *p* < 0.01; rs = 0.14, *p* < 0.01, respectively). Additionally, social media use was positively correlated with both thin- and muscular-ideal internalization (rs = 0.13, *p* < 0.01 and rs = 0.26, *p* < 0.01, respectively). Among both girls and boys, the correlations between variables were generally small, except for a strong correlation between thin-ideal internalization and body dissatisfaction observed in girls (β = 0.600, *p* < 0.001) [26].

It is also worth noting the differences in online activity between the sexes. Girls were more likely to indicate social media as their main form of online activity, while boys preferred games and informational content. This distinction may partly explain why girls are more vulnerable to a decline in self-esteem, as the platforms they use are more saturated with visual content based on aesthetic norms and social exposure.

In light of the above findings, it seems extremely important to develop educational programs aimed at building a critical approach to media content, strengthening emotional resilience, and promoting healthy digital habits. Particular attention should be paid to girls, who, as this study shows, are more susceptible to the negative consequences of excessive presence in digital visual environments. It is also necessary to further deepen our knowledge about the role of online content—future studies should more precisely differentiate between types of media (e.g., influencer accounts, fitness content, beauty guides) that may have varying effects on the psyche of young people.

The findings of this study extend beyond theoretical contributions and hold important implications for public health, education, and clinical practice. For schools, the results suggest the value of embedding digital literacy and critical media awareness modules into curricula to help adolescents recognize and resist harmful appearance-based comparisons. Clinicians and counselors working with youth can integrate discussions about healthy digital habits into therapeutic settings, particularly with adolescent girls who may be most vulnerable to the adverse effects of prolonged online engagement. Policymakers may also draw on these findings to guide collaborations with technology companies, encouraging the implementation of safeguards that limit exposure to harmful content and promote positive digital environments.

## 6. Strengths and Limitations

One of the key strengths of the present study is the use of a three-point measurement design (T1, T2, T3), which allowed for the minimization of random mood fluctuations and enhanced the reliability and validity of the psychological constructs measured. Averaging scores across three time points contributed to greater temporal stability and increased the credibility of the obtained data. Another notable advantage is the relatively large sample size (N = 500) and the nearly balanced gender distribution (approximately 1:1), which ensured adequate statistical power and improved the generalizability of the findings to the broader population of late adolescents. Furthermore, the study employed well-validated and widely used psychometric instruments BES and SES. Both scales demonstrated high internal consistency in the current sample (Cronbach’s α > 0.85) and satisfactory temporal stability, confirming their suitability for repeated assessment. The use of the CAWI (Computer-Assisted Web Interviewing) method facilitated convenient and anonymous participation, likely increasing the honesty of self-reported responses. The assignment of individual identification codes enabled the linkage of data across time points while maintaining full anonymity and GDPR compliance. Additionally, the statistical models accounted for key confounding variables (e.g., BMI, gender, time spent online), which further strengthens the internal validity of the findings.

Despite its strengths, several important limitations should be considered when interpreting the results. First and foremost, the reliance on self-report methodology introduces potential biases such as cognitive distortions (e.g., social desirability bias), imprecise estimation of Internet usage, and perceptual inaccuracies in evaluating one’s own body. A further limitation lies in the lack of data regarding the type of online content consumed. As a result, it remains unclear whether the observed reductions in body esteem and self-esteem are driven by overall screen time or specific exposure to content such as social media, influencers, or appearance-related messaging. Future research should incorporate more granular measures to address this gap. It is also important to acknowledge that, although the analyses controlled for variables such as gender and BMI, other potentially influential factors were not assessed. These include levels of physical activity, parenting style, social support, and mental health status-all of which may contribute to body image and self-esteem. The absence of these variables limits the comprehensiveness of the explanatory model. Another key limitation concerns the sampling method, which was based on availability and voluntary participation. Individuals who opted into an online study may differ systematically from the general adolescent population in terms of motivation, engagement, and Internet use habits. This introduces the potential for volunteer bias, which may limit the external validity of the findings. Moreover, although the repeated-measures design provided consistency over time, it is not possible to fully exclude the possibility that some participants consulted others or modified their responses between measurement waves. Given that the study was conducted in home environments, full control over the testing conditions was not feasible. Finally, due to the cross-sectional-longitudinal design without experimental manipulation, the observed relationships are correlational in nature. Thus, causal inferences cannot be definitively drawn regarding the directionality of the associations identified.

While the present study offers robust insights into the relationship between digital media use and adolescent self-perception, several avenues remain open for further research. Future studies should investigate the role of specific content types (e.g., influencer-driven beauty content, fitness-oriented media, educational platforms) in shaping body esteem and self-esteem. Longitudinal and experimental designs could help clarify causal pathways between digital exposure and psychological outcomes. Moreover, integrating additional psychosocial variables—such as family dynamics, peer support, or individual coping strategies—would contribute to a more comprehensive explanatory model. Such work may also inform the development of more targeted, evidence-based interventions that account for gender-specific vulnerabilities.

## 7. Conclusions

The present study provides clear evidence that adolescent girls aged 15–18 years old experience significantly lower body esteem and global self-esteem compared to their male peers. These differences are not explained by objective body metrics such as BMI, which showed no significant association with psychological outcomes. Instead, time spent online emerged as a key predictor, with longer daily Internet use, use-especially beyond 4 h, linked to lower scores on both the BES and the SES. The negative effects of digital exposure were particularly evident among girls. These findings underscore the importance of environmental and perceptual factors, such as digital content and social comparison, over biological determinants in shaping adolescents’ psychological well-being. They highlight the need to address the role of online environments in reinforcing appearance-related pressures, especially for adolescent girls who may be more vulnerable to such influences. Interventions that foster media literacy and emotional resilience are crucial in mitigating these risks.

A practical implication is the development of school-based programs focused on digital hygiene and critical media engagement. Structured educational modules that include discussions on body image, social media influence, and healthy online behavior, along with parent involvement, may help reduce the negative impact of digital overexposure. Such programs should be gender-sensitive and embedded in broader mental health promotion strategies aimed at supporting adolescents’ self-esteem and body acceptance in the digital age.

From a public health standpoint, the findings support the implementation of digital hygiene curricula in secondary education, including modules on social media literacy, emotional regulation, and resilience-building. Collaborations with tech platforms to moderate appearance-centric content and promote algorithmic transparency could further mitigate harm, especially for vulnerable subgroups such as adolescent girls.

## Figures and Tables

**Table 1:** Characteristics of the participants (N = 500).

Group	Female (*n* = 251)	Male (*n* = 249)	*p*-Value
Average age (SD)	16.5 (1.1)	16.4 (1.2)	NS
Average BMI (SD)	22.0 (3.1)	22.2 (3.0)	NS
Online time > 4 h	54%	39%	0.01

NS—nonsignificance.

**Table 2:** Percentage of participants with low body acceptance and low self-esteem * (N = 500).

Group (Gender/Online Time)		% Low BES	% Low SES
Female	<2 h	18.0%	11.0%
	2–4 h	29.6%	22.2%
	4–6 h	41.8%	34.1%
	>6 h	40.0%	32.0%
Male	<2 h	12.5%	7.1%
	2–4 h	18.1%	10.2%
	4–6 h	16.2%	13.7%
	>6 h	20.0%	14.0%

* The thresholds adopted were BES < 40 (low body acceptance) and SES < 20 (low self-esteem).

**Table 3:** Multiple regression model of BES and SES predictors (N = 500).

Variable		β	SE	t	*p*	Interpretation
BES	Gender	−4.57	0.90	−5.1	0.001	Strong negative correlation— females on average scored 4.57 points less
	BMI	−0.11	0.15	−0.72	0.472	NS
	Online time	−0.56	0.22	−2.52	0.012	Negative correlation— more time, lower score
SES	Gender	−2.22	0.35	−6.31	0.001	Strong negative correlation— females on average scored 2.22 points less
	BMI	0.02	0.06	0.40	0.687	NS
	Online time	−0.40	0.09	−4.58	0.001	Strong negative correlation— more time, lower score

Note. β = standardized regression coefficient; SE = standard error; t = t-value; *p* = probability value (significance level); NS—nonsignificance.

## Data Availability

Data are contained within the article.
